# General psychopathology, internalising and externalising in children and functional outcomes in late adolescence

**DOI:** 10.1111/jcpp.13067

**Published:** 2019-05-02

**Authors:** Hannah Sallis, Eszter Szekely, Alexander Neumann, Alexia Jolicoeur‐Martineau, Marinus van IJzendoorn, Manon Hillegers, Celia M.T. Greenwood, Michael J Meaney, Meir Steiner, Henning Tiemeier, Ashley Wazana, Rebecca M. Pearson, Jonathan Evans

**Affiliations:** ^1^ Centre for Academic Mental Health, Population Health Sciences Bristol Medical School University of Bristol Bristol UK; ^2^ MRC Integrative Epidemiology Unit University of Bristol Bristol UK; ^3^ UK Centre for Tobacco and Alcohol Studies School of Psychological Science University of Bristol Bristol UK; ^4^ Department of Psychiatry Faculty of Medicine McGill University Montréal QC Canada; ^5^ Lady Davis Institute for Medical Research Jewish General Hospital Montréal QC Canada; ^6^ Department of Child and Adolescent Psychiatry/Psychology Erasmus MC University Medical Center Rotterdam Rotterdam The Netherlands; ^7^ Department of Psychology, Education and Child Studies Erasmus University Rotterdam Rotterdam The Netherlands; ^8^ Primary Care Unit School of Clinical Medicine University of Cambridge Cambridge UK; ^9^ Department of Epidemiology, Biostatistics and Occupational Health McGill University Montréal QC Canada; ^10^ Departments of Oncology and Human Genetics McGill University Montréal QC Canada; ^11^ Douglas Mental Health University Institute Montréal QC Canada; ^12^ Sackler Program for Epigenetics & Psychobiology McGill University Montréal QC Canada; ^13^ Singapore Institute for Clinical Sciences Singapore City Singapore; ^14^ Women's Health Concerns Clinic St. Joseph's Healthcare Hamilton ON Canada; ^15^ Departments of Psychiatry & Behavioural Neurosciences and Obstetrics & Gynecology McMaster University Hamilton ON Canada; ^16^ Department of Social and Behavioral Sciences Harvard T. H. Chan School of Public Health Boston MA USA; ^17^ Centre for Child Development and Mental Health Jewish General Hospital Montréal QC Canada

**Keywords:** Childhood psychopathology, Avon Longitudinal Study of Parents and Children, Maternal Adversity, Vulnerability and Neurodevelopment, Generation Rotterdam, developmental pathways

## Abstract

**Background:**

Internalising and externalising problems commonly co‐occur in childhood. Yet, few developmental models describing the structure of child psychopathology appropriately account for this comorbidity. We evaluate a model of childhood psychopathology that separates the unique and shared contribution of individual psychological symptoms into specific internalising, externalising and general psychopathology factors and assess how these general and specific factors predict long‐term outcomes concerning criminal behaviour, academic achievement and affective symptoms in three independent cohorts.

**Methods:**

Data were drawn from independent birth cohorts (Avon Longitudinal Study of Parents and Children (ALSPAC), *N* = 11,612; Generation R, *N* = 7,946; Maternal Adversity, Vulnerability and Neurodevelopment (MAVAN), *N* = 408). Child psychopathology was assessed between 4 and 8 years using a range of diagnostic and questionnaire‐based measures, and multiple informants. First, structural equation models were used to assess the fit of hypothesised models of shared and unique components of psychopathology in all cohorts. Once the model was chosen, linear/logistic regressions were used to investigate whether these factors were associated with important outcomes such as criminal behaviour, academic achievement and well‐being from late adolescence/early adulthood.

**Results:**

The model that included specific factors for internalising/externalising and a general psychopathology factor capturing variance shared between symptoms regardless of their classification fits well for all of the cohorts. As hypothesised, general psychopathology factor scores were predictive of all outcomes of later functioning, while specific internalising factor scores predicted later internalising outcomes. Specific externalising factor scores, capturing variance not shared by any other psychological symptoms, were not predictive of later outcomes.

**Conclusions:**

Early symptoms of psychopathology carry information that is syndrome‐specific as well as indicative of general vulnerability and the informant reporting on the child. The ‘general psychopathology factor' might be more relevant for long‐term outcomes than specific symptoms. These findings emphasise the importance of considering the co‐occurrence of common internalising and externalising problems in childhood when considering long‐term impact.

## Introduction

Psychiatric diagnostic nosology reflects efforts to delineate specific criteria for diagnosing distinct mental disorders across the life span. With each revised edition of the diagnostic criteria (American Psychiatric Association, 2013; World Health Organisation, 1993), the total number of disorders as well as the number of diagnoses received by each individual is rising, both for children and adults (Insel, 2014). As the set of possible diagnoses expands, there is an increasing amount of symptom overlap between diagnoses. A similar story is seen within self‐ and parent‐reported questionnaires for internalising and externalising symptoms, where scales are strongly correlated. Therefore, it is important to understand what this comorbidity and common variance of childhood psychological symptoms represent and its relevance for later functioning. Our current research question is whether there is a general factor of child psychopathology and if so, does this general factor predict important outcomes in later life?

While childhood psychopathology is traditionally grouped into internalising and externalising disorders, there remains considerable comorbidity between these two categories (Angold, Costello, & Erkanli, 1999). In addition, the stability of these categories over time is unclear (Murray, Eisner, & Ribeaud, 2016; Rutter, Kim‐Cohen, & Maughan, 2006; Shevlin, McElroy, & Murphy, 2017). It is common for underlying internalising disorders to manifest as behavioural problems usually attributed to externalising disorders and vice versa, for example, a child could exhibit features of conduct disorder which result from being anxious (Bubier & Drabick, 2009). This complexity of the relationship between internalising and externalising symptoms can make it difficult to categorise childhood psychopathology, determine aetiology, investigate outcomes and plan interventions.

Understanding the overlap between internalising and externalising symptoms as well the contribution of multiple informants may improve the characterisation and predictive models of childhood psychopathology. This objective is important for improving childhood problems and preventing later adverse outcomes (Vigo, Thornicroft, & Atun, 2016). Early identification of those at risk is essential for prevention strategies.

Structural equation models (SEM) enable us to consider both general psychopathology and more specific dimensions within the same model (Caspi et al., 2014; Laceulle, Vollebergh, & Ormel, 2015; Lahey et al., 2012; Neumann et al., 2016). In this framework, each symptom can both contribute variance that is shared with other symptoms *and* that is unique to itself. The underlying assumption of bifactor SEM models is that the shared variance amongst items represents a common construct (in our case general psychopathology), and simultaneously unique variance to a smaller cluster of items represents more specific constructs (for example specific externalising and internalising behaviours). This approach differs from other techniques such as network analysis, which conceptualise psychopathology as a group of interlinked symptoms without any underlying construct.

When comparing bifactor models to alternative models, rather than simply relying on model fit statistics which can be fallible in these situations, models should be assessed in terms of their criterion validity, scientific and clinical utility (Bonifay, Lane, & Reise, 2017; Lahey, Krueger, Rathouz, Waldman, & Zald, 2017). To this end, we evaluate the fit of a bifactor model of child psychopathology using data from three independent birth cohorts. We subsequently investigate the prognostic utility of this model by testing the association between childhood psychopathology and later behavioural, educational and psychological outcomes in adolescence and early adulthood. Given the comorbidity between internalising and externalising problems and little evidence of stability of these categories over time, we hypothesise that the general psychopathology factor will be associated with a range of outcomes. However, specific internalising symptoms will be associated only with psychological symptoms and specific externalising with behavioural outcomes.

## Methods

### Studies and measures

Data used for these analyses were drawn from the Developmental Research in Environmental Adversity, Mental health, BIological susceptibility and Gender (DREAM BIG ‐ http://www.dreambigresearch.com) consortium formed in 2016 to investigate the association between prenatal adversity and later childhood mental health outcomes. DREAM BIG consists of 4 prenatal population cohorts: the Avon Longitudinal Study of Parents and Children (ALSPAC; Boyd et al., 2013; Fraser et al., 2013), the Generation Rotterdam (Generation R) Study (Kooijman et al., 2016; Tiemeier et al., 2012), the Maternal Adversity, Vulnerability and Neurodevelopment (MAVAN) project (O'Donnell et al., 2014) and the Growing Up in Singapore Towards healthy Outcomes (GUSTO) study (Soh et al., 2014). A full description of each cohort can be found in the relevant cohort profiles and in Appendix S1 in the Supporting Information. Given that in GUSTO collection of data relevant to the present analysis is still ongoing due to the young age of participants, it was not included in the present study.

Each cohort has collected several measures capturing mental health during early childhood. In the development of a GPF, we focused on those symptoms that quantify internalising and externalising symptoms. Measures included the Development and Well‐Being Assessment, Strengths and Difficulties Questionnaire and the Child Behaviour Checklist. A complete list of measures and full details of each are provided in Appendix S2.

To maximise the number of participants included in the models and prevent sampling bias, missing information was imputed for participants with available data on at least one psychopathology subscale. Further details on imputation strategies are outlined in Appendix S3. Within ALSPAC, sensitivity analyses were also performed on the subset of participants with complete data on all subscales.

### Modelling psychopathology in childhood

Measures relating to psychopathology from 4 to 8 years of age were collated. Single measures of each subscale were used for ALSPAC and Generation R, while repeated measures of Child Behaviour Checklist, Strengths and Difficulties Questionnaire and Conners' Parent Rating Scale were used in the MAVAN study. These included self‐, parental‐, teacher‐ and observer‐rated measures (Table S1).

Confirmatory factor analysis, a subset of SEM, was used to estimate the general structure of psychopathology, based on previous studies, including one report also based on a subset of data from the Generation R cohort (Lahey et al., 2015; Neumann et al., 2016). We used a stepwise approach to construct a model of childhood psychopathology, beginning with a simple unifactor model and building up to a more complex bifactor structure (see Tables 1 and S5 for a complete overview). Model fit was evaluated in each cohort using several model fit indices: root mean square error of approximation (RMSEA), comparative fit index (CFI) and Tucker–Lewis index (TLI). CFI and TLI represent the fit compared to a null model with no correlations, adjusted for model complexity. In the case of the TLI, we can interpret the value as percentage of fit improvement compared with the null model. RMSEA is an absolute measure of fit, again adjusted for model complexity. When investigating model fit, RMSEA values of <.05 (Browne & Cudeck, 1992) and CFI/TLI values of >.9 (Hooper, Coughlan, & Mullen, 2008) are generally used to indicate good fit.

**Table 1:** Model fit statistics for final model of childhood psychopathology

	ALSPAC			Generation R			MAVAN		
	RMSEA (90% CI)	CFI	TLI	RMSEA (90% CI)	CFI	TLI	RMSEA (90% CI)	CFI	TLI
Unifactor	.083 (.079, .087)	.297	.274	.103 (.102, .104)	.544	.509	.084 (.082, .086)	.460	.440
Internalising & externalising	.082 (.078, .086)	.311	.289	.124 (.123, .126)	.324	.287	.082 (.079, .084)	.544	.526
Bifactor – internalising, externalising, rater and GPF	.036 (.036, .036)	.876	.863	.048 (.047, .049)	.915	.894	.055 (.052, .057)	.787	.763

ALSPAC, Avon Longitudinal Study of Parents and Children; CFI, comparative fit index; MAVAN, Maternal Adversity, Vulnerability and Neurodevelopment; TLI, Tucker–Lewis index; RMSEA, root mean square error of approximation.

Individual items were first loaded onto a single factor to investigate whether items appeared to be measuring a single construct (unifactor structure). Subsequent models separated the items into specific internalising/externalising factors, defined a priori, to explore whether the items were capturing these two distinct constructs. Most item‐scale allocations were known; the few items that did not have a pre‐existing allocation, (e.g. the fieldworker‐rated behaviour items in ALSPAC), two researchers independently assigned them based on a priori knowledge (to either the internalising or externalising factor). Although most items loaded strongly onto the factors to which they were initially assigned, some items were moved if modification indices from the initial model indicated that items would be a better fit on the alternative factor (a list of these modifications can be found in the footnote to Table S2).

We also investigated whether additionally accounting for variance common to a specific informant by adding so‐called ‘reporter' factors (i.e. mother, father, teacher, child or fieldworker) would further improve model fit (Table 1).

**Figure 1:**
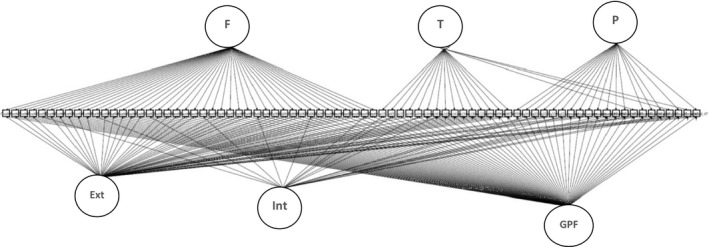
Model of childhood psychopathology at age 7. F, T and P are the ‘methods' factors corresponding to the observer who rated each item. F: Fieldworker‐rated items; T: Teacher‐rated items; P: parent‐rated items. Int, Ext and GPF correspond to the specific internalising, specific externalising and general psychopathology factors. A complete list of the items loading onto each factor can be found in Table S2

In the final bifactor model, each item loaded onto the GPF, a reporter factor, and its corresponding specific factor (i.e. internalising/externalising) with a few exceptions [with the exception of the SDQ prosocial score, the Social and Communication Disorders Checklist (SCDC), the sleep and ‘other' sum scores of the Childhood Behaviour Checklist (CBCL), the thought and social problems subscales of the Teacher Report Form (TRF) and the Social Responsiveness Scale (SRS)]. The final model solution is displayed in Figure 1 and Tables S2–S4. Factors in the final model were defined to be orthogonal.

Analyses were performed using MPlus v.7 in ALSPAC and the lavaan R package in MAVAN, and Generation R. Robust maximum likelihood (MLR) estimators were used in the MAVAN and Generation R cohorts, while weighted least square means and variances (WLSMV) were used in ALSPAC. Latent variables were standardised in each of the cohorts.

### Testing the associations between general and specific factors in the bifactor model and long‐term outcomes

The bifactor model was tested by examining the associations between the general psychopathology, specific internalising and specific externalising factors with later outcomes measured in ALSPAC in early adulthood (Figure S1). These associations were compared with internalising and externalising symptoms in a model without general psychopathology (see Figure S2).

Outcomes included the following: (a) diagnoses of depression and anxiety at 18 years assessed using the Revised Clinical Interview Schedule (CIS‐R), (b) psychological well‐being assessed at age 21 using the Warwick‐Edinburgh Mental Wellbeing Scale (WEMWBS), (c) criminal activity (defined as any self‐reported involvement with the police) at age 21; (d) alcohol use (defined as any problem drinking) assessed by the Alcohol Use Disorders Identification Test (AUDIT) at age 21 (e) and educational attainment as indicated by receiving a pass grade (C or above) at English or mathematics at GCSE (public examinations taken at age 16 in the United Kingdom).

Analyses were run using an unadjusted model in addition to a model adjusting for child gender, maternal age at delivery, maternal education and income. These were chosen a priori as measures of adversity that could act as confounders. These were variables that are associated with child internalising/externalising symptoms and the later outcomes but not part of the causal pathway.

## Results

A full description of each of the cohorts can be found in the cohort profiles (Boyd et al., 2013; Fraser et al., 2013; Jaddoe et al., 2006; O'Donnell et al., 2014; Soh et al., 2014). The final sample size for analysis was 408 in MAVAN, 7,946 in Generation R and 11,612 in ALSPAC.

### Modelling childhood psychopathology

The unifactor model in each cohort had a poor fit, as did the model with internalising and externalising factors only. Model fit improved with the addition of rater factors and further improved with the inclusion of the GPF. Consistently across all cohorts, the best fitting model was a bifactor solution containing a GPF, specific internalising/externalising factors and rater factors. Model fit statistics for all models tested are shown in Tables 1 and S5.

Initially, the correlation between the internalising and externalising factors was constrained to zero in all models. As a sensitivity analysis, these factors were allowed to correlate. In none of the cohorts, did this substantially improve model fit and the correlation between the internalising–externalising factors was small. Consequently, to ensure consistent and parsimonious models, the final bifactor models in all cohorts were constrained as orthogonal.

The final model structure for ALSPAC, MAVAN and Generation R are displayed in Figure 1 and Tables S2–S4.

### Sensitivity analysis

1,129 (9.7%) participants in the ALSPAC cohort had complete data on all items included in the psychopathology model. Analyses were rerun in ALSPAC restricting to this subset of complete cases. A similar pattern was observed, with a bifactor model containing a GPF, specific internalising/externalising factors and observer factors found to be the best solution (Table S6).

### Testing the associations between general and specific factors in the bifactor model and long‐term outcomes

Results showed that the general psychopathology was associated with a range of different outcomes (Table 2). Specifically, there was an association between the GPF and developing a depressive disorder (β* *= .117, *p* = .001), experiencing decreased psychological well‐being at age 21 (β* *= −.062, *p* = .001) and failing mathematics (β* *= −.235, *p* < .001) or English GCSE at age 16 (β = −.260, *p* < .001). Unexpectedly, there was an association between GPF and reduced risk of problem drinking (β* *= −.102, *p* < .001) but no association with criminal activity and none with anxiety. In the same bifactor model, the specific internalising factor was associated with increased risk for depression (β* *= .085, *p* = .030) and anxiety (β* *= .184, *p* < .001), decreased well‐being (β* *= −.089, *p* < .001) and failure at mathematics GCSE (β* *= −.054, *p* = .017). There was little association with later problem drinking, criminal behaviour or English GCSE results. There was no association between the specific externalising factor scores from the bifactor model and adverse outcomes but some association with a lower risk for later problem drinking (β* *= −.080, *p* = .010) and better performance at both mathematics (β* *= .050, *p* = .055) and English GCSE (β* *= .082, *p* = .001).

**Table 2:** Association between childhood psychopathology and later outcomes adjusted for maternal age at delivery, maternal education, household income and child gender

	Factor	*N*	INT/EXT model (no GPF)		Bifactor model (INT, EXT, GPF)	
			Estimate	*p*‐value	Estimate	*p*‐value
Depressive disorder	INT	4,260	.106	.013	.085	.030
	EXT		.145	<.001	−.027	.497
	GPF		–	–	.117	.001
Anxiety	INT	4,260	.204	<.001	.184	<.001
	EXT		.085	.063	−.064	.147
	GPF		–	–	.069	.080
Well‐being	INT	4,205	−.100	<.001	−.089	<.001
	EXT		−.079	<.001	−.025	.267
	GPF		–	–	−.062	.001
Problem drinking	INT	3,654	−.054	.065	−.040	.158
	EXT		−.114	<.001	−.080	.010
	GPF		–	–	−.102	<.001
Crime	INT	3,684	−.017	.641	−.022	.529
	EXT		.073	.035	.062	.075
	GPF		–	–	.050	.085
Mathematics GCSE – pass grade (C or above)	INT	6,081	−.097	<.001	−.054	.017
	EXT		−.308	<.001	.050	.055
	GPF		–	–	−.235	<.001
English GCSE – pass grade (C or above)	INT	6,201	−.032	.294	.015	.533
	EXT		−.383	<.001	.082	.001
	GPF		–	–	−.260	<.001

In contrast when not including the GPF in the model, the externalising factor was associated with increased criminality, depression, anxiety, failure at both mathematics and English GCSE, decreased well‐being and lower problem drinking (Table 2). The internalising factor showed similar associations with depression, anxiety, well‐being and reduced attainment in mathematics. These associations were stronger in the absence of a general psychopathology factor.

Full results for the adjusted models are presented in Table 2 and for the unadjusted models in Table S7.

## Discussion

Here, we systematically evaluated the structure of childhood psychological symptoms in three birth cohorts in the international DREAM BIG consortium. In each cohort, this bifactor model included a specific internalising and specific externalising factor, as well as a general psychopathology factor representing variance common to all psychological symptoms.

Having evaluated this bifactor model structure across three cohorts, we were able to examine the extent to which this factor was associated with long‐term follow‐up data from ALSPAC. As hypothesised, the GPF was associated with a range of outcomes, including mathematics and English GCSE scores which support the criterion validity of this general factor. However, the specific internalising factor still predicted depression, anxiety and well‐being when accounting for general psychopathology. In contrast, the specific externalising factor which showed some associations in the simpler model was no longer predictive of adverse outcomes once general psychopathology was taken into account.

This suggests that *shared* variance between externalising and internalising symptoms may be more important for long‐term outcomes than specific externalising symptoms. However, these results should be replicated in independent cohorts. If this finding does hold, this does not imply that externalising symptoms are not associated with later functioning, rather, that once the shared variance between externalising and internalising is taken into account (i.e. in the form of the GPF), the remaining unique variance does not relate to the examined outcomes of adolescent/adult functioning. This finding is consistent with those of Brikell and colleagues who investigated the association between a general psychopathology factor model and genetic risk scores for attention‐deficit hyperactivity disorder (Brikell et al., 2018). This is also in line with findings from Patalay and colleagues who found an association between a general psychopathology factor and educational outcomes in an adolescent sample (Patalay et al., 2015).

Simply put, the shared variance in the GPF represents children having both externalising and internalising symptoms and the specific factors representing children with ‘residual' symptoms. Thus, our results suggest that those at greater risk of later adverse outcomes such as poor school performance are likely to present with *both* internalising and externalising symptoms. Identifying these children would enrich our understanding of the developmental pathways which could inform intervention or prevention strategies, such as the development of a universal therapy or repurposing existing therapies in a transdiagnostic approach (Caspi & Moffitt, 2018; Krueger & Eaton, 2015). Our results also highlighted the importance of accounting for variation common to a specific informant, as this further improved model fit in each cohort. This partially reflects the individual differences inherent in how different informants answer specific items, but it also reflects the fact that raters generally complete entire questionnaires. Thus, the different rater factors also likely captured questionnaire‐specific variance. In sum, the informant does have a unique contribution to the child's symptom scores, which is important to account for in data analysis.

There are a number of limitations to our analysis that should be considered. First, the measures of psychopathology partially differed across the cohorts and child self‐reports were unavailable in ALSPAC for this age group. However, each cohort used a broad range of measures to capture childhood psychopathology and a comparable model solution was found to be the best across all cohorts. Second, there were missing data in each cohort. In order to maximise power and reduce sampling bias, we imputed missing data for all participants with available observations on at least one psychopathology subscale. Importantly, consistent results emerged in the sensitivity analysis conducted in the ALSPAC subset of complete cases only. We did not impute outcomes in ALSPAC so were unable to check how estimates from our prediction models compared with those from imputed data. However, when running these prediction models in the subset of complete cases for the bifactor model, the pattern of results remained largely consistent, albeit it with lower power to detect effects within this sample. Third, different statistical programmes and imputation strategies were used across the cohorts; however, our conclusions about which was the best model were consistent despite these differences. Finally, these analyses were based on data from convenient time points in all cohorts thus do not inform us regarding the trajectory of symptoms of internalising and externalising disorders over time. However, we were able to identify a comparable factor structure of early childhood psychopathology across three independent cohorts. A strength of this study is that this new consortium provides an exceptional opportunity to test similar hypotheses across comparable cohorts harmonised across major constructs, a unique strength which addresses key concerns of replication in our field (Open Science Collaboration, 2015).

## Conclusion

We suggest that models of childhood psychopathology should account for the co‐occurrence of internalising and externalising symptoms, as well as variance specific to these symptoms, and the informant reporting on the child. Our findings further indicate that this co‐occurrence of externalising and internalising symptoms may be more informative for the prevention of long‐term adverse outcomes than specific symptoms. However, this finding should be replicated in further studies.

**Key points**

- Internalising and externalising symptoms are common in childhood and impact on social and educational functioning as well as influencing future health outcomes.
- We used data from three diverse international birth cohorts to evaluate a model of childhood psychopathology which accounts for both shared and specific variation.
- The general psychopathology factor predicted a range of adverse outcomes, while the specific internalising factor specifically predicted later internalising problems.
- Our findings suggest that *shared* variance between externalising and internalising items is important for long‐term outcomes.
- This could suggest interventions should focus on co‐occurrence of symptoms in order to prevent long‐term impact.

## Supporting information


**Appendix S1.** Studies.
**Appendix S2.** Measures of childhood psychopathology.
**Appendix S3.** Imputation strategy.
**Table S1.** Summary of measures across cohorts.
**Table S2.** Structure of the bifactor model constructed for the ALSPAC cohort.
**Table S3.** Structure of the bifactor model constructed for the Generation R cohort.
**Table S4.** Structure of the bifactor model constructed for the MAVAN cohort.
**Table S5.** Model fit statistics for final model of childhood psychopathology.
**Table S6.** Model fit statistics restricting to complete cases in the ALSPAC cohort.
**Table S7.** Unadjusted association between childhood psychopathology and later outcomes.
**Figure S1.** Association between childhood psychopathology factors and later outcomes in ALSPAC.
**Figure S2.** Association between childhood internalising and externalising factors with later outcomes in ALSPAC.
