# Supplementary material for: General psychopathology, internalising and externalising in children and functional outcomes in late adolescence
Source: J Child Psychol Psychiatry. 2019 May 2;60(11):1183–90. doi: 10.1111/jcpp.13067 (PMC6849715; doi:10.1111/jcpp.13067)
Supplement: Supplementary file 1 — Appendix S1. Studies. Appendix S2. Measures of childhood psychopathology. Appendix S3. Imputation strategy. Table S1. Summary of measures across cohorts. Table S2. Structure of the bifactor model constructed for the ALSPAC cohort. Table S3. Structure of the bifactor model constructed for the Generation R cohort. Table S4. Structure of the bifactor model constructed for the MAVAN cohort. Table S5. Model fit statistics for final model of childhood psychopathology. Table S6. Model fit statistics restricting to complete cases in the ALSPAC cohort. Table S7. Unadjusted association between childhood psychopathology and later outcomes. Figure S1. Association between childhood psychopathology factors and later outcomes in ALSPAC. Figure S2. Association between childhood internalising and externalising factors with later outcomes in ALSPAC. [file JCPP-60-1183-s001.docx]

**Supplementary info**

**Supplementary materials and methods**

**Studies**

*Avon Longitudinal Study of Parents and Children (ALSPAC)*

ALSPAC is a longitudinal pregnancy cohort which aimed to recruit all pregnant women in the former county of Avon with an expected due date between April 1991 and December 1992. Detailed information has continued to be collected on mothers, partners and children in the cohort, this process has been described in detail elsewhere (Boyd et al., 2013; Fraser et al., 2013). Out of the 14,541 mothers who entered the study, 11,612 children had data available on at least one psychopathology subscale at age 7 years. Ethical approval for the study was obtained from the ALSPAC Ethics and Law Committee and the Local Research Ethics Committees. A fully searchable data dictionary with information on all available measures is available at <http://www.bris.ac.uk/alspac/researchers/data-access/data-dictionary/>.

*Generation Rotterdam (Generation R)*

Generation R is a population-based birth cohort with the aim to identify early environmental and genetic determinants of development and health (Kooijman et al., 2016; Tiemeier et al., 2012). Mothers living in Rotterdam and delivery date between April 2002 and January 2006 were eligible for the study. Out of the 9901 children who entered the study, 7946 had information on at least one psychopathology subscale available at ages 6-8. All analyses are based on this sample. Parents gave informed consent for their children's participation. The Generation R Study is conducted in accordance with the World Medical Association Declaration of Helsinki and study protocols have been approved by the Medical Ethics Committee of the Erasmus Medical Centre, Rotterdam.

*Maternal Adversity, Vulnerability and Neurodevelopment study (MAVAN)*

The Maternal Adversity, Vulnerability and Neurodevelopment (MAVAN) study is a Canadian community-based birth cohort. Pregnant women were recruited from obstetric clinics in hospitals from Montreal (Quebec) and Hamilton (Ontario) if they were 18 years of age or older and fluent in either French or English. Greater details about the cohort are provided elsewhere (O’Donnell et al., 2014). Ethical approval for this study was obtained from the Douglas Mental Health University Institute (Montreal) and Saint Joseph’s Hospital (Hamilton). Of the 590 mothers who entered the study, 408 had children on which information on at least one psychopathology subscale was available at ages 4-6 years.

**Measures of childhood psychopathology**

***Development and Well-Being Assessment (DAWBA).*** The Development and Well-Being Assessment (DAWBA) is a combination of questionnaires, interviews and ratings, designed to be administered by nonclinical interviewers to generate ICD-10 and DSM-IV psychiatric diagnoses (Goodman, Ford, Richards, Gatward, & Meltzer, 2000). The DAWBA was designed for use in samples of 5-16 year olds. The DAWBA was rated by parents and teachers in the ALSPAC cohort at age 7 years.

***Social and Communication Disorders Checklist (SCDC).*** The Social and Communication Disorders Checklist (SCDC) is a 12-item screening tool for autistic traits/developmental disorders (Skuse et al., 1997). The SCDC is a parent-reported measure ranging from 0 to 24, with higher scores indicative of more autistic traits. 9 of the items measure traits relating to social interaction and communication skills, with the remaining 3 items measuring behavioural problems and functional impairment. Parents are asked to rate each statement according to behaviour in the previous 6 months as ‘not true’, ‘quite or sometimes true’ or ‘very or often true’, with corresponding scores of 0, 1 and 2. The SCDC was rated by parents in the ALSPAC cohort when children were aged 7.5 years.

***Additional teacher questions.*** Additional questions were included within the teacher rated questionnaires in ALSPAC which assessed the number of troublesome and awkward behaviours, attention and activity, and the burden of these behaviours on the child. These were measured when the children were around 7 years of age.

***Field worker rated observations.*** ALSPAC participants attended a clinic at age 7, at which they completed the following 7 sessions: coordination, hearing, allergy, biological samples, measurements and body statistics, vision, and word skills. After each of these sessions, the field worker was asked to rate the child on each of the following attributes: cooperative, shy, fidget, active, attention and responsive/rapport. Each attribute was measured on a scale of 1-3, for example 1 = cooperative, 2 = somewhat cooperative, 3 = uncooperative.

***Social Responsiveness Scale (SRS).*** Autistic-like traits were measured using a validated short-form of the SRS (Constantino et al., 2003). (Constantino et al., 2003). The primary caregiver (91% mothers) rated autistic-like traits when children were 6 years (M=6.2, SD=0.5). The subscales Social Cognition, Social Communication, and Autistic Mannerism were calculated.

***Teachers Rating Form (TRF).*** At age 7 years (M=6.7, SD=1.3) teachers assessed child psychological problems with the TRF 6-18 (Thomas M Achenbach & Rescorla, 2001), which includes the subscales: Anxious/ Depressed, Withdrawn/Depressed, Somatic Complaints, Social Problems, Thought Problems, Attention Problems, Rule-Breaking Behaviour, and Aggressive Behaviour.

***Berkeley Puppet Interview (BPI).*** Self-reported behaviour problems were measured in Generation R using the BPI, a semi-structured interactive interview(Arseneault et al., 2003) con (Arseneault et al., 2003) conducted at age 6 years (M=6.2, SD=0.5). During the interview two hand puppets made opposite statements and the child had to choose which statement fit them best. Scoring was performed with video tapes with high intercoder reliability (Ringoot et al., 2013).ility (Ringoot et al., 2013). The BPI consists of six subscales: Depression, Separation Anxiety, Overanxious, Oppositional Defiant, Overt Hostility, and Conduct Problems.

***Child Behaviour Checklist (CBCL).*** The CBCL (T.M. Achenbach & Rescorla, 2000)(T.M. Achenbach & Rescorla, 2000) was was used to obtain standardized parent reports of common internalising and externalising symptoms. For the current study, we included the seven empirically derived narrowband syndrome scales of the CBCL: Emotionally Reactive, Anxious/Depressed, Somatic Complaints, Withdrawn, Sleep Problems, Attention Problems and Aggressive Behaviour. Items were rated on a 3-point scale (not true, somewhat/sometimes true and very/often true). Within MAVAN, the CBCL was completed by the mother at two time points, i.e., at age 4 and 5 years. In Generation R, questionnaires were completed by the primary caregiver (92% mothers) when children were on average 6 years (M=6.1, SD=0.5).

***Strengths and Difficulties Questionnaire (SDQ).*** The SDQ is a reliable and valid brief measure of prosocial behaviour and psychopathology in children (Goodman, 2001). The SDQ asks about 25 attributes of the child, both positive and negative. Each item can be marked as ‘not true’, ‘somewhat true’ or ‘certainly true’. The measure comprises five subscales, each with five items: emotional symptoms, conduct problems, hyperactivity-inattention, peer problems and prosocial behaviour. The MAVAN cohort included maternal ratings of the SDQ at ages 5 and 6 years and paternal ratings at 5 years. Within Generation R, the same collection of questionnaires that contained the CBCL, were used to assess items from the SDQ prosocial behaviour scale. The SDQ was also assessed in ALSPAC, with parent and teacher ratings collected at around 7 years of age.

***Conners’ Parent Rating Scale–Revised: Short Form (CPRS-R).*** Within the MAVAN study, ratings of children’s externalizing problems were measured using both maternal and paternal ratings on the CRPS-R:S (Conners, Sitarenios, Parker, & Epstein, 1998) at two time points: at five and six years of age. In Generation R, the CPRS-R was completed by the primary caregiver (90% mothers) when children were 8 years (M=8.2, SD=0.2). The CPRS-R is a well-validated questionnaire for the assessment of ADHD and ODD. Items were rated on a 4-point scale from 0 (not true at all) to 3 (very much true). Three scales of the CPRS-R were used: Inattention/Cognitive Problems, Hyperactivity and Oppositional.

***The Preschool Age Psychiatric Assessment (PAPA).*** The PAPA is a semi-structured researcher-administered diagnostic parent interview, feasible and validated for children under age 7 (Zeanah et al., 2009). Instead of diagnosis, we used the number of symptoms mothers reported for the disorders assessed by the PAPA. These were separation anxiety, generalized anxiety disorder, phobias, social phobia, overanxious, panic disorder, ADHD, CD, ODD, and combined depression and dysthymia.

***The Pictorial Dominic Questionnaire***. The Dominic is a highly structured, developmentally sensitive self-report measure of DSM-IV-TR diagnoses for school-aged children (6-11 years) (Valla, Bergeron, Bidaut-Russell, St-Georges, & Gaudet, 1997). Participants reported on the presence or absence (yes/no) of symptoms of seven disorders with the help of standardized drawings. The symptom scales include Major Depressive Disorder, Generalized Anxiety Disorder, Specific Phobias, Social Phobia, ADHD, ODD, and CD. The present study did not include children’s ratings for the ADHD subscale, given its low reliability at this young age according to the manual (Valla, Bergeron, & Smolla, 2000).

**Imputation strategy**

For the ALSPAC cohort, imputation was performed using the ice command implemented in Stata 14. Variables included in the imputation model included each variable in the P-factor, plus all earlier measures of these variables and auxiliary variables deemed to be related to missingness. Auxiliary variables included maternal and paternal socioeconomic status and level of education, maternal and paternal alcohol use, marital status, perinatal depression, drug use, domestic violence, partner affection and aggression, gestational age, maternal age at delivery, ethnicity, gestation at enrolment, child temperament. 40 imputed datasets were created, and the parameter estimates for each imputation were combined using Rubin’s rules as applied by the ‘imputation’ package in Mplus.

In MAVAN and Generation R, missing data points were estimated using the full information maximum likelihood (FIML) function specified within lavaan. This function uses all available data on the subscales that were included in the model to estimate missing values.

**References**

Achenbach, T. M., & Rescorla, L. A. (2000). Manual for the ASEBA Preschool Forms and Profiles. *Burlington VT: University of Vermont, Research Center for Children, Youth, & Families.*

Achenbach, T. M., & Rescorla, L. A. (2001). *Manual for the ASEBA School-Age Forms & Profiles*. Burlington VT: University of Vermont, Research Center for Children, Youth, & Families.

Arseneault, L., Moffitt, T. E., Caspi, A., Taylor, A., Rijsdijk, F. V, Jaffee, S. R., … Measelle, J. R. (2003). Strong genetic effects on cross-situational antisocial behaviour among 5-year-old children according to mothers, teachers, examiner-observers, and twins’ self-reports. *Journal of Child Psychology and Psychiatry*, *44*(6), 832–848.

Boyd, A., Golding, J., Macleod, J., Lawlor, D. A., Fraser, A., Henderson, J., … Davey Smith, G. (2013). Cohort Profile: the ’children of the 90s’--the index offspring of the Avon Longitudinal Study of Parents and Children. *Int J Epidemiol*, *42*(1), 111–127. https://doi.org/10.1093/ije/dys064

Conners, C. K., Sitarenios, G., Parker, J. D., & Epstein, J. N. (1998). The revised Conners’ Parent Rating Scale (CPRS-R): factor structure, reliability, and criterion validity. *Journal of Abnormal Child Psychology*, *26*(4), 257–268.

Constantino, J. N., Davis, S. A., Todd, R. D., Schindler, M. K., Gross, M. M., Brophy, S. L., … Reich, W. (2003). Validation of a Brief Quantitative Measure of Autistic Traits: Comparison of the Social Responsiveness Scale with the Autism Diagnostic Interview-Revised. *Journal of Autism and Developmental Disorders*, *33*(4), 427–433. https://doi.org/10.1023/A:1025014929212

Fraser, A., Macdonald-Wallis, C., Tilling, K., Boyd, A., Golding, J., Davey Smith, G., … Lawlor, D. A. (2013). Cohort Profile: the Avon Longitudinal Study of Parents and Children: ALSPAC mothers cohort. *Int J Epidemiol*, *42*(1), 97–110. https://doi.org/10.1093/ije/dys066

Goodman, R. (2001). Psychometric Properties of the Strengths and Difficulties Questionnaire. *Journal of the American Academy of Child & Adolescent Psychiatry*, *40*(11), 1337–1345. https://doi.org/10.1097/00004583-200111000-00015

Goodman, R., Ford, T., Richards, H., Gatward, R., & Meltzer, H. (2000). The Development and Well-Being Assessment: description and initial validation of an integrated assessment of child and adolescent psychopathology. *J Child Psychol Psychiatry*, *41*(5), 645–655.

Kooijman, M. N., Kruithof, C. J., van Duijn, C. M., Duijts, L., Franco, O. H., van IJzendoorn, M. H., … Jaddoe, V. W. V. (2016). The Generation R Study: design and cohort update 2017. *European Journal of Epidemiology*, *31*(12), 1243–1264. https://doi.org/10.1007/s10654-016-0224-9

O’Donnell, K. A., Gaudreau, H., Colalillo, S., Steiner, M., Atkinson, L., Moss, E., … MAVAN Research Team. (2014). The maternal adversity, vulnerability and neurodevelopment project: theory and methodology. *Canadian Journal of Psychiatry. Revue Canadienne de Psychiatrie*, *59*(9), 497–508. https://doi.org/10.1177/070674371405900906

Ringoot, A. P., Jansen, P. W., Steenweg-de Graaff, J., Measelle, J. R., van der Ende, J., Raat, H., … Tiemeier, H. (2013). Young children’s self-reported emotional, behavioral, and peer problems: The Berkeley Puppet Interview. *Psychological Assessment*, *25*(4), 1273–1285. https://doi.org/10.1037/a0033976

Skuse, D. H., James, R. S., Bishop, D. V. M., Coppin, B., Dalton, P., Aamodt-Leeper, G., … Jacobs, P. A. (1997). Evidence from Turner’s syndrome of an imprinted X-linked locus affecting cognitive function. *Nature*, *387*(6634), 705–708. https://doi.org/10.1038/42706

Tiemeier, H., Velders, F. P., Szekely, E., Roza, S. J., Dieleman, G., Jaddoe, V. W. V., … Verhulst, F. C. (2012). The Generation R Study: A Review of Design, Findings to Date, and a Study of the 5-HTTLPR by Environmental Interaction From Fetal Life Onward. *Journal of the American Academy of Child & Adolescent Psychiatry*, *51*(11), 1119–1135.e7. https://doi.org/10.1016/j.jaac.2012.08.021

Valla, J., Bergeron, L., Bidaut-Russell, M., St-Georges, M., & Gaudet, N. (1997). Reliability of the Dominic-R: a young child mental health questionnaire combining visual and auditory stimuli. *Journal of Child Psychology and Psychiatry*, *38*(6), 717–724.

Valla, J., Bergeron, L., & Smolla, N. (2000). The Dominic-R: A Pictorial Interview for 6- to 11-Year-Old Children. *Journal of the American Academy of Child & Adolescent Psychiatry*, *39*(1), 85–93. https://doi.org/10.1097/00004583-200001000-00020

Zeanah, C. H., Egger, H. L., Smyke, A. T., Nelson, C. A., Fox, N. A., Marshall, P. J., & Guthrie, D. (2009). Institutional Rearing and Psychiatric Disorders in Romanian Preschool Children. *American Journal of Psychiatry*, *166*(7), 777–785. https://doi.org/10.1176/appi.ajp.2009.08091438

**Table S1. Summary of measures across cohorts**

|  | **ALSPAC** | | **Gen R** | | **MAVAN** | |
| --- | --- | --- | --- | --- | --- | --- |
| **Rater** | **Measure**  **(Age)** | **Subscale** | **Measure**  **(Age)** | **Subscale** | **Measure**  **(Age)** | **Subscale** |
| Parent | **DAWBA**  (7 years) | Depression | **CBCL**  (6 years) | Emotionally reactive | **CBCL**  (Mother – 4 and 6 years) | Emotionally reactive |
|  |  | General anxiety |  | Anxious/depressed |  | Anxious/depressed |
|  |  | Separation anxiety |  | Somatic complaints |  | Somatic complaints |
|  |  | Social phobia |  | Withdrawn |  | Withdrawn |
|  |  | Specific phobia |  | Sleep problems |  | Sleep problems |
|  |  | ADHD |  | Attention problems |  | Attention problems |
|  |  | Conduct disorder |  | Aggressive behaviour |  | Aggressive behaviour |
|  |  | ODD |  | Sum score of other items |  |  |
|  | **SDQ**  (7 years) | Emotional problems |  |  | **SDQ**  (Mother – 5 and 6 years; Father – 5 years) | Emotional problems |
|  |  | Peer problems |  |  |  | Peer problems |
|  |  | Conduct problems |  |  |  | Conduct problems |
|  |  | Hyperactivity |  |  |  | Hyperactivity |
|  |  | Prosocial |  |  |  | Prosocial |
|  |  |  | **CPRS-R**  (8 years) | ADHD inattentive | **CPRS-R**  (Mother and father – 5 and 6 years) | ADHD inattentive |
|  |  |  |  | ADHD hyperactive impulsive |  | ADHD hyperactive impulsive |
|  |  |  |  | ODD |  | ODD |
|  |  |  | **SRS**  (6 years) | Social cognition | **PAPA**  (Mother – 6 years) | Separation anxiety |
|  |  |  |  | Social communication |  | GAD |
|  |  |  |  | Autistic mannerism |  | Social phobia |
|  | **SCDC**  (7.5 years) | - |  |  |  | Overanxious disorder |
|  |  |  |  |  |  | Panic disorder |
|  |  |  |  |  |  | Depression & dysthymia |
|  |  |  |  |  |  | ADHD |
|  |  |  |  |  |  | CD |
|  |  |  |  |  |  | ODD |
| Teacher | **DAWBA**  (7 years) | ADHD | **TRF**  (7 years) | Anxious/depressed |  |  |
|  |  | Conduct disorder |  | Withdrawn/depressed |  |  |
|  |  | ODD |  | Somatic complaints |  |  |
|  | **SDQ**  (7 years) | Emotional problems |  | Social problems |  |  |
|  |  | Peer problems |  | Thought problems |  |  |
|  |  | Conduct problems |  | Attention problems |  |  |
|  |  | Hyperactivity |  | Rule-breaking behaviour |  |  |
|  |  | Prosocial |  | Aggressive behaviour |  |  |
|  | **Additional questions**  (7 years) | Activity symptoms score |  |  |  |  |
|  |  | Attention symptoms score |  |  |  |  |
|  |  | Burden of attention/activity |  |  |  |  |
|  |  | Awkward behaviours score |  |  |  |  |
|  |  | Troublesome behaviours |  |  |  |  |
|  |  | Burden of troublesome behaviours |  |  |  |  |
| Field worker | **Field worker observations**  (7 years) | Cooperative |  |  |  |  |
|  |  | Fidget |  |  |  |  |
|  |  | Active |  |  |  |  |
|  |  | Attention |  |  |  |  |
|  |  | Responsive |  |  |  |  |
| Child |  |  | **BPI**  (6 years) | Depression | **Dominic**  (6 years) | Depression |
|  |  |  |  | Separation anxiety |  | Separation anxiety |
|  |  |  |  | Overanxious |  | Overanxious |
|  |  |  |  | Oppositional defiant |  | Oppositional defiant |
|  |  |  |  | Overt hostility |  | Conduct disorder |
|  |  |  |  | Conduct problems |  | Phobias |

DAWBA – Development and Well-Being Assessment; ADHD – Attention deficit hyperactivity disorder; ODD – oppositional defiant disorder; ^1^ CBCL - Child behaviour checklist; SDQ – Strengths and Difficulties Questionnaire; SCDC - Social and Communication Disorders Checklist; CPRS-R - Conners’ parent rating scale – revised: short-form; ^3^ SRS - Social responsiveness scale; TRF – Teachers rating form; PAPA – Preschool Age Psychiatric Assessment; BPI – Berkeley puppet interview

Table S2. Structure of the bifactor model constructed for the ALSPAC cohort

| **Internalising factor** | | | **Externalising factor** | | | **General factor (GPF)** |
| --- | --- | --- | --- | --- | --- | --- |
| **Rater** | **Measure** | **Scale** | **Rater** | **Measure** | **Scale** |  |
| Parent | DAWBA | Depression | Parent | DAWBA | ADHD | All items plus |
|  |  | General anxiety |  |  | Conduct disorder | SDQ parent and |
|  |  | Separation anxiety |  |  | Oppositional defiant disorder | teacher rated |
|  |  | Social phobia |  | SDQ | Conduct problems | prosocial |
|  | SDQ | Emotional problems |  |  | Hyperactivity | scores, and |
|  |  |  |  |  | Peer problems | parent rated |
| Teacher | SDQ | Emotional problems | Teacher | DAWBA | ADHD | SCDC score |
|  |  |  |  |  | Conduct disorder |  |
|  |  |  |  |  | Oppositional defiant disorder |  |
|  |  |  |  | SDQ | Conduct problems |  |
|  |  |  |  |  | Hyperactivity |  |
|  |  |  |  |  | Peer problems |  |
|  |  |  |  | Additional questions | Activity symptoms score |  |
|  |  |  |  |  | Attention symptoms score |  |
|  |  |  |  |  | Burden of attention/activity |  |
|  |  |  |  |  | Awkward behaviours score |  |
|  |  |  |  |  | Troublesome behaviours |  |
|  |  |  |  |  | Burden of troublesome behaviours |  |
|  |  |  | Field-worker |  | Cooperative |  |
|  |  |  |  |  | Fidget |  |
|  |  |  |  |  | Active |  |
|  |  |  |  |  | Attention |  |
|  |  |  |  |  | Responsive |  |

In the initial model, items on the SDQ peer problems subscale were split across internalising and externalising factors, the prosocial subscale was included on the externalising factor. Field worker rated ‘responsive’ items were included on the internalising factors and the ‘shyness’ items were included in the model. In the final model, peer problems were included as a single subscale on the externalising factor, the prosocial subscales are included on the GPF only and responsiveness has been moved to the externalising factor. Shyness items have been removed from the model as these were not found to load strongly on any of the factors.

Table S3. Structure of the bifactor model constructed for the Generation R cohort

| **Internalising factor** | | | **Externalising factor** | | | **General factor (GPF)** |
| --- | --- | --- | --- | --- | --- | --- |
| **Rater** | **Measure** | **Scale** | **Rater** | **Measure** | **Scale** |  |
| Parent | CBCL | Emotionally reactive | Parent | CBCL | Attention problems | All subscales plus CBCL sleep problems, CBCL sum score of other items, TRF social problems, TRF thought problems, and SRS subscales |
|  |  | Anxious/depressed |  |  | Aggressive behaviour |  |
|  |  | Somatic complaints |  |  |  |  |
|  |  | Withdrawn |  |  |  |  |
|  |  |  |  | CPRS-R | ADHD inattentive |  |
|  |  |  |  |  | ADHD hyperactive impulsive |  |
|  |  |  |  |  | ODD |  |
| Teacher | TRF | Anxious/depressed | Teacher | TRF | Attention problems |  |
|  |  | Withdrawn/depressed |  |  | Rule-breaking behaviour |  |
|  |  | Somatic complaints |  |  | Aggressive behaviour |  |
| Child |  | Depression | Child | BPI | Oppositional defiant |  |
|  |  | Separation anxiety |  |  | Overt hostility |  |
|  |  |  |  |  | Conduct problems |  |

Table S4. Structure of the bifactor model constructed for the MAVAN cohort

| **Internalising factor** | | | **Externalising factor** | | | **General factor (GPF)** |
| --- | --- | --- | --- | --- | --- | --- |
| **Rater** | **Measure** | **Scale** | **Rater** | **Measure** | **Scale** |  |
| Mother | CBCL | Emotionally reactive | Mother | CBCL | Attention problems | All items plus CBCL mother-rated sleep problems and SDQ mother-rated and father-rated prosocial behaviour |
|  |  | Anxious/depressed |  |  | Aggressive behaviour |  |
|  |  | Withdrawn |  | PAPA | ADHD |  |
|  |  | Somatic problems |  |  | Oppositional defiant disorder |  |
|  | PAPA | Social phobia |  |  | Conduct disorder |  |
|  |  | Overanxious | Mother and father | SDQ | Conduct problems |  |
|  |  | Panic |  |  | Hyperactivity |  |
|  |  | Depression |  |  | Peer problems |  |
| Mother and father | SDQ | Emotional problems |  | Conner’s PRS-R | Oppositional problems |  |
| Child | Dominic | Separation anxiety |  |  | Inattention/cognitive problems |  |
|  |  | Overanxious |  |  | Hyperactivity |  |
|  |  | Simple phobias | Child | Dominic | Oppositional defiant disorder |  |
|  |  | Depression |  |  | Conduct disorder |  |

Table S5. Model fit statistics for final model of childhood psychopathology

|  | **ALSPAC** | | | **Generation R** | | | **MAVAN** | | |
| --- | --- | --- | --- | --- | --- | --- | --- | --- | --- |
|  | RMSEA  (90% CI) | CFI | TLI | RMSEA  (90% CI) | CFI | TLI | RMSEA  (90% CI) | CFI | TLI |
| Unifactor | 0.083  (0.079, 0.087) | 0.297 | 0.274 | 0.103  (0.102, 0.104) | 0.544 | 0.509 | 0.084  (0.082, 0.086) | 0.460 | 0.440 |
| Internalising & externalising | 0.082  (0.078, 0.086) | 0.311 | 0.289 | 0.124  (0.123, 0.126) | 0.324 | 0.287 | 0.082  (0.079, 0.084) | 0.544 | 0.526 |
| Internalising & externalising (correlated)* | 0.086  (0.080, 0.084) | 0.243 | 0.218 | 0.105  (0.103, 0.105) | 0.352 | 0.315 | 0.081  (0.078, 0.083) | 0.559 | 0.541 |
| Internalising, externalising & rater | 0.047  (0.043, 0.050) | 0.778 | 0.763 | 0.060  (0.058, 0.061) | 0.857 | 0.836 | 0.061  (0.059, 0.064) | 0.754 | 0.733 |
| Internalising, externalising & rater (correlated)** | 0.050  (0.046, 0.054) | 0.752 | 0.735 | 0.060  (0.058, 0.061) | 0.857 | 0.836 | 0.061  (0.059, 0.064) | 0.754 | 0.733 |
| Bifactor – internalising, externalising & GPF | 0.060  (0.056, 0.064) | 0.643 | 0.619 | 0.090  (0.089, 0.091) | 0.674 | 0.629 | 0.072  (0.069, 0.074) | 0.620 | 0.592 |
| Bifactor – internalising, externalising, rater & GPF | 0.036  (0.036, 0.036) | 0.876 | 0.863 | 0.048  (0.047, 0.049) | 0.915 | 0.894 | 0.055  (0.052, 0.057) | 0.787 | 0.763 |
| Correlated bifactor – internalising, externalising, rater & GPF*** | 0.036  (0.036, 0.036) | 0.872 | 0.859 | 0.048  (0.047, 0.049) | 0.915 | 0.894 | 0.055  (0.052, 0.057) | 0.787 | 0.762 |

* Correlation between internalising and externalising factors: ALSPAC=0.286, p<0.001; Generation R=0.664, p<0.001; MAVAN=0.572, p<0.001

** Correlation between internalising and externalising factors: ALSPAC=0.284, p<0.001; Generation R=0.108, p=0.002; MAVAN=-0.026, p=0.814

*** Correlation between internalising and externalising factors: ALSPAC=-0.102, p<0.001; Generation R=-0.230, p<0.001; MAVAN=-0.051, p=0.578

Table S6. Model fit statistics restricting to complete cases in the ALSPAC cohort

|  | **ALSPAC** | | |
| --- | --- | --- | --- |
|  | RMSEA | CFI | TLI |
| Unifactor | 0.066 (0.064, 0.067) | 0.436 | 0.418 |
| Rater | 0.051 (0.049, 0.052) | 0.665 | 0.653 |
| Instrument | 0.069 (0.068, 0.070) | 0.370 | 0.349 |
| Internalising & externalising | 0.066 (0.065, 0.067) | 0.422 | 0.404 |
| Internalising, externalising & rater | 0.040 (0.039, 0.041) | 0.800 | 0.787 |
| Bifactor – internalising, externalising & P-factor | 0.042 (0.041, 0.043) | 0.774 | 0.759 |
| Bifactor – internalising, externalising, rater & P-factor | 0.026 (0.025, 0.028) | 0.915 | 0.906 |
| Correlated bifactor – internalising, externalising, rater & P-factor* | 0.026 (0.025, 0.028) | 0.914 | 0.905 |

* Correlation = -0.157, p<0.001

Table S7. Unadjusted association between childhood psychopathology and later outcomes

|  |  |  |  | No GPF (unadjusted) | | | Bifactor model  (unadjusted) | |
| --- | --- | --- | --- | --- | --- | --- | --- | --- |
|  |  | Factor | N | Estimate | P-value | Estimate | | P-value |
| Depressive disorder | | INT | 4260 | 0.120 | 0.004 | 0.108 | | 0.006 |
|  |  | EXT |  | 0.085 | 0.041 | 0.007 | | 0.865 |
|  |  | GPF |  | - | - | 0.063 | | 0.076 |
| Anxiety |  | INT | 4260 | 0.214 | <0.001 | 0.200 | | <0.001 |
|  |  | EXT |  | 0.036 | 0.433 | -0.039 | | 0.383 |
|  |  | GPF |  | - | - | 0.022 | | 0.572 |
| Wellbeing | | INT | 4205 | -0.108 | <0.001 | -0.101 | | <0.001 |
|  |  | EXT |  | -0.041 | 0.066 | -0.047 | | 0.042 |
|  |  | GPF |  | - | - | -0.028 | | 0.145 |
| Problem drinking | | INT | 3654 | -0.065 | 0.029 | -0.053 | | 0.067 |
|  | | EXT |  | -0.108 | 0.0001 | -0.068 | | 0.037 |
|  | | GPF |  | - | - | -0.093 | | 0.001 |
| Crime | | INT | 3684 | -0.042 | 0.267 | -0.056 | | 0.114 |
|  |  | EXT |  | 0.180 | <0.001 | 0.032 | | 0.383 |
|  |  | GPF |  | - | - | 0.143 | | <0.001 |
| Maths GCSE – pass grade (C or above) | | INT | 6081 | 0.079 | 0.004 | -0.038 | | 0.112 |
|  |  | EXT |  | 0.352 | <0.001 | 0.040 | | 0.148 |
|  | | GPF |  | - | - | -0.269 | | <0.001 |
| English GCSE – pass grade (C or above) | | INT | 6201 | 0.004 | 0.876 | 0.041 | | 0.095 |
|  |  | EXT |  | 0.447 | <0.001 | 0.090 | | 0.001 |
|  | | GPF |  | - | - | -0.350 | | <0.001 |

Figure S1. Association between childhood psychopathology factors and later outcomes in ALSPAC

MDD

Figure S2. Association between childhood internalising and externalising factors with later outcomes in ALSPAC

MDD
